# Psychiatric Comorbidities of Inflammatory Bowel Disease: It Is a Matter of Microglia’s Gut Feeling

**DOI:** 10.3390/cells13020177

**Published:** 2024-01-17

**Authors:** Gohar Fakhfouri, Nataša R. Mijailović, Reza Rahimian

**Affiliations:** 1 Department of Psychiatry, Douglas Hospital, McGill University, Montreal, QC H4H 1R3, Canada; ghazal.fakhfouri@mail.mcgill.ca; 2 Department of Pharmacy, Faculty of Medical Sciences, University of Kragujevac, 34000 Kragujevac, Serbia; nacakg@gmail.com; 3 McGill Group for Suicide Studies, Douglas Mental Health Institute, McGill University, 6875 Boulevard LaSalle, Montreal, QC H4H 1R3, Canada

**Keywords:** microglia, IBD, gut–brain axis, psychiatric disorders, neuroinflammation, gut microbiota, innate immunity

## Abstract

Inflammatory bowel disease (IBD), a common term for Crohn’s disease and ulcerative colitis, is a chronic, relapse-remitting condition of the gastrointestinal tract that is increasing worldwide. Psychiatric comorbidities, including depression and anxiety, are more prevalent in IBD patients than in healthy individuals. Evidence suggests that varying levels of neuroinflammation might underlie these states in IBD patients. Within this context, microglia are the crucial non-neural cells in the brain responsible for innate immune responses following inflammatory insults. Alterations in microglia’s functions, such as secretory profile, phagocytic activity, and synaptic pruning, might play significant roles in mediating psychiatric manifestations of IBD. In this review, we discuss the role played by microglia in IBD-associated comorbidities.

## 1. Introduction

Inflammatory bowel disease (IBD), an umbrella term for Crohn’s disease (CD) and ulcerative colitis (UC), is a chronic and relapsing condition with a complex etiology [1]. While the exact cause of IBD remains elusive, it is well established that CD and UC arise from the intricate interplay of genetic, environmental, and microbial factors, accompanied by immune system abnormalities [2,3]. The most frequent intestinal manifestations of IBD include abdominal pain, diarrhea, rectal bleeding, and malnutrition. IBD is linked to a range of extra-intestinal complications. Several reports confirm that psychiatric comorbidities are prevalent in IBD patients, with depression rates that are three times higher compared to the general population [4,5,6]. Similarly, the rates of sleep disturbances, mood disorders, and psychological distress are higher among IBD patients, even during periods of remission [7]. Cognitive functions are also impaired in IBD patients [8]. Unfortunately, these comorbidities significantly affect the quality of life in these patients and are often poorly understood and controlled. Bipolar disorder and schizophrenia are also more frequent in IBD patients compared to healthy control subjects [9,10]. Yet, anxiety and depression remain the dominant psychiatric comorbidities in IBD patients. The prevalence of depressive disorders in IBD patients varies from 21% to 25%, while anxiety disorders are present in 19.1% to 35% of these patients [11]. Compared with the general population, IBD patients are twice as likely to have an affective disorder [12]. A study by Panara et al. showed that depression is independently associated with the female gender, the active form of a disease, and a more aggressive disease course [13]. The severity of IBD has been found to correlate with emotional factors and the personal perception of the disease, denoting IBD as a psychosomatic disorder [14]. The diagnosis of IBD itself significantly affects the well-being of the individual, while the unpredictable and chronic nature of the disease can result in additional challenges, such as social isolation, stigmatization, and feelings of shame [15]. Furthermore, individuals with IBD may be more susceptible to the impacts of stress [16]. Recognizing the psychological distress in the management of IBD is crucial, as it not only influences the patient’s quality of life but is also linked to increased disease activity, increased relapse frequency, and the greater utilization of healthcare services [17,18].

The gut–brain axis represents a bidirectional communication network that comprises neural, hormonal, immunological, and microbial signals [19]. This well-established gut–brain connection plays a crucial role in the pathogenesis of IBD. Signals from the gut allow the brain to control the physiological and inflammatory status of the gut [20]. It has been shown that P-selectin-mediated monocyte–cerebral endothelium adhesive interactions can link peripheral organ inflammation with sickness behaviors [21]. It is well known that stress exacerbates IBD, and it can independently reactivate experimental colitis [22,23]. In a pioneer study, Ghia et al. reported that impaired vagal nerve (VN) function increases susceptibility to IBD in a murine model of depression [24]. Furthermore, they demonstrated a crucial role for macrophage in linking depression and its susceptibility to intestinal inflammation via the VN, demonstrating that the macrophage Alpha7 nicotinic acetylcholine receptor (α7 nAChR) is involved in the depression-induced reactivation of dormant colitis [25]. In addition, the effects of gut inflammation on the brain’s innate immune responses have been studied. In this context, it has been shown that central nervous system (CNS) excitability is altered via the activation of microglia and the brain production of inflammatory cytokines following gut inflammation [26].

Neuroinflammation is implicated in the pathophysiology of depression, along with other psychiatric disorders [27]. Microglia, the brain’s innate immune cells, are crucially involved in different neuroinflammatory processes [28]. More recent findings revealed that microglia are functionally more complex than previously considered [29]. Indeed, in response to inflammatory insults, microglia can transform into reactive states and adopt different transcriptional and functional features [30,31,32,33]. These changes depend on the nature of the inflammatory insult, brain region, sex, and age [34,35,36]. A rapidly growing literature suggests that psychiatric disorders, particularly major depressive disorder (MDD), can be associated with chronic low-level neuroinflammation and long-lasting priming and the sensitization of microglia [37,38].

In this article, we discuss the pivotal role of microglia in IBD-associated psychiatric disorders and deal with different important phenomena that can be affected by IBD, such as the microbiota–gut–brain axis, microglial synaptic pruning, neurogenesis, and blood–brain barrier (BBB) integrity. Furthermore, the alterations of microglia’s metabolic pathways under inflammatory conditions are discussed. We also point out how microglia fine-tuning may ultimately guide us in designing new therapeutics for the treatment of psychiatric complications in IBD patients.

## 2. Gut Microbiota Abnormalities in IBD and Its Possible Connection to the Brain’s Innate Immune Response

**Figure 1:**
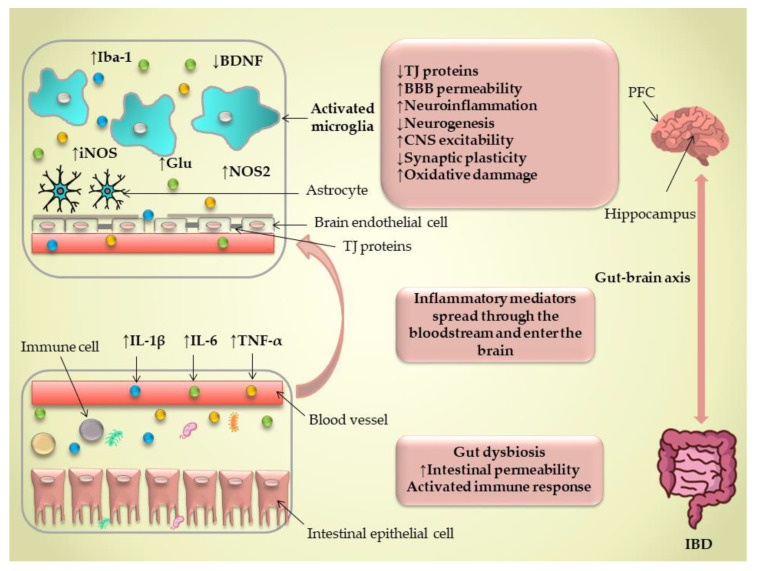
Schematic overview of the neuroinflammatory changes observed in IBD, leading to behavioral alterations. Intestinal inflammation in IBD induced by gut dysbiosis leads to impaired intestinal barrier integrity, activation of immune cells, and consequential production of pro-inflammatory cytokines. These inflammatory mediators enter the bloodstream and increase blood–brain barrier (BBB) permeability by interrupting tight junction (TJ) physiology, thus facilitating the entry of pro-inflammatory mediators into brain parenchyma and causing microglial activation. Activated microglia further exacerbate the inflammatory response in the CNS followed by increased IL-1β, IL-6, and TNF-α levels, as well as increasing iNOS and nitrite levels in brain tissue. Activated microglia leads to impairments in neurogenesis, oxidative status, CNS excitability, and synaptic plasticity, thus contributing to behavioral abnormalities. ↑—increased; ↓—decreased.

It is established that the gut–brain axis’s communication is bidirectional and that the gut microbiome and its composition play a dynamic role in regulating the different physiological functions of the CNS. This complex communication occurs through immune, enteric, and neural pathways [39,40]. New cutting-edge technologies in RNA sequencing and omics analyses point to an imbalance in the composition and function of the intestinal microbiome in the pathogenesis of IBD, known as dysbiosis [41]. Dysbiosis in IBD includes the diminished diversity of microbiota; an increase in pathogenic bacteria, mucolytic bacteria, and sulfate-reducing bacteria; and a reduction in short-chain fatty acid (SCFA)-producing bacteria. These changes disturb the host immune system and barrier integrity, leading to chronic inflammation and abnormal immune responses [41,42] (Figure 1).

The gut microbiota plays a significant role in the microglia’s development and functioning. The abrogation or reduction in microbiota in germ-free or antibiotic-treated mice is associated with abnormalities in gene expression profiles and morphological properties, as well as an immature phenotype of microglia, leading to damaged innate immune responses [43,44,45] and impaired fear extinction learning [46]. The genes that display distinct expression patterns in microglia are especially abundant in pathways associated with the organization and formation of synapses. This suggests that targeted manipulation of the microbiota could potentially modify the way microglia participate in synaptic pruning and influence the remodeling of dendritic spines, since these alterations may contribute to behavioral abnormalities [43,46]. The impact of the microbiome on microglia displays temporal and sexual dimorphism [47]. Furthermore, microbiota-derived metabolites, such as SCFA acetate, butyrate, and propionate; aryl hydrocarbon receptor (AhR) ligands [48,49]; lipopolysaccharides (LPSs) [50]; and also the newly discovered quorum sensing peptides [51], have been recently denoted as bacterium-derived molecules that are able to modulate microglia. A part of the crosstalk between gut microbiota and the CNS is mediated by the VN, and any alteration in the status of intestinal inflammation is conveyed to the CNS through vagal afferents. This ultimately influences microglial activation and the level of neuroinflammation [40,52]. Gut dysbiosis causes significant changes in the peripheral myeloid cell population, impaired immune function, and microglial activation, affecting synaptic transmission and plasticity and behaviorally relevant network activities in the hippocampus [53]. Dysbiosis-induced behavioral changes are also accompanied by alterations in the brain-derived neurotrophic factor and its receptor, tropomyosin receptor kinase B signaling, transient receptor potential vanilloid subtype 1 phosphorylation, and neuronal activity in the hippocampus [54].

Salvo and colleagues demonstrated that dextran sulfate sodium (DSS)-induced colitis at weaning causes microbiota–gut–brain axis deficits in adulthood [55]. While the acute inflammatory response was resolved, cognitive deficits and anxiety-like behavior remained in adult mice. These behavioral changes were accompanied by neuroinflammation, impaired neurogenesis, and the increased hippocampal expression of ionized calcium binding adaptor molecule 1 (Iba-1), interleukin (IL)-1 beta, and inducible nitric oxide synthase (iNOS), along with gut microbiota changes [55]. Other compelling research suggests that the disruption of the microbiota–gut–brain axis in IBD might not always co-occur with neuroinflammation and the activation of microglia. Vicentini et al. demonstrated increased anxiety-like behavior, changes in gut microbiota, and increased central tumor necrosis factor (TNF) expression. Upon transferring cecal contents from colitic mice into either germ-free or antibiotics-treated mice, the recipient mice displayed comparable behavioral alterations, but without any signs of colonic or neuroinflammation [56].

These studies suggest the importance of gut microbiota alterations in driving behavioral abnormalities in colitis. It is yet unclear to what extent and in what manner a disrupted microbiota can modify microglial function, consequently impacting the onset and progression of these changes. Gaining a deeper understanding of the enduring consequences and pinpointing the specific bacteria and their metabolites will facilitate the discovery of potential therapeutic targets.

## 3. Blood–Brain Barrier Integrity in IBD

IBD-associated peripheral inflammation can impact BBB permeability by interrupting tight junction (TJ) physiology in brain endothelial cells [57]. Several investigations have shown that circulating cytokines modulate the expression of TJ proteins in endothelial cells [58,59,60,61], while pro-inflammatory and anti-inflammatory cytokines elicit differential roles. For example, IL-1β has been shown to downregulate TJ proteins, such as claudin-5 (Cld-5) and zonula occludens (ZO)-1, via modulating astrocytic sonic hedgehog (SHH) production [61]. Conversely, in vivo, IL-10 reduced inflammation severity, diminished increased BBB permeability by inhibiting the apoptosis of brain microvascular endothelial cells, and improved Cld-5 expression in these cells [62]. The cytokine-induced dysregulation of BBB has been also reported in animal models of colitis. Increased IL-6 levels led to the reduced expression of occludin and Cld-5 in the hippocampus and cortex of colitis-afflicted mice [63]. A significant increase in BBB permeability, particularly in the circumventricular organs within the brain parenchyma, was detected using the trinitrobenzene sulphonic acid (TNBS) model of colitis [64].

The choroid plexus (ChP), a densely vascularized tissue that produces cerebrospinal fluid (CSF) and does not possess a BBB, is a critical juncture where peripheral and central immune responses intersect [65]. The gene expression patterns of pro-inflammatory cytokines, immune cells, and factors involved in immune cell trafficking are dysregulated in the ChP of depressed suicides [65]. Carloni et al. pointed out new mechanisms that underlie gut–brain communication in IBD models [66]. Their findings indicated that intestinal inflammation drives gut vasculature barrier impairment and the recruitment of inflammatory cells to the brain, alters brain permeability, and leads to an altered microglial phenotype, resulting in short-term memory deficits and anxiety-like behavior. They demonstrated a region-specific remodeling of BBB adapter TJ proteins, such as a decrease in the expression of ZO-1 in the somatosensory cortical area and the paraventricular nucleus of the thalamus, while no differences for Cld-5 were observed. Furthermore, they observed the closure of the vascular barrier in the ChP as a defensive strategy against spreading the inflammation from the gut [66]. These results pointed out the role of gut–brain vascular axis dysregulation in mental disorders observed in colitis.

It should be mentioned that BBB disruption following inflammation is very context-dependent and is not a common phenomenon in animal models of depression [32]. The chronic social stress disrupted the integrity of the BBB by reducing the expression of the Cld-5, facilitated peripheral IL-6 passage across the BBB, and induced depressive-like behavior in male mice [67]. Hence, it seems that peripheral inflammation following exposure to stress or gut inflammation might affect BBB integrity. However, brain region, sex, type of stressor, and inflammatory insult are important factors that influence BBB integrity. Namely, Menard et al. reported BBB leakage only in the nucleus accumbens, an important part of the ventral striatum, of chronically, socially stressed male mice but not in other brain regions and stress models [67].

It is noteworthy that alongside circulating cytokines and chemokines, microglia activation might affect the permeability of BBB in IBD. Alterations in the intercommunication between endothelial cells, neurons, and microglia, are associated with a wide range of inflammation-related brain disorders, particularly where the integrity of the BBB is compromised [68]. Increasing evidence indicates that activated microglia modulate the expression of TJs. Conversely, the endothelium can modulate the activation of microglia [69]. Activated microglia produce enzymes that generate reactive oxygen species (ROS) that lead to degeneration in oligodendrocytes and increase BBB permeability by reducing the expression of vascular endothelial cadherin, occludin, and Cld-5 in the microvascular endothelial cells (ECs) [69]. Microglia have dual effects on BBB permeability induced by systemic inflammation [70]. LPS-induced inflammation triggers the migration of microglia toward blood vessels in the initial stage, protecting BBB integrity by increasing the expression of Cld-5 and establishing direct connections with ECs. However, during prolonged inflammation, microglia transform into a CD68-expressing phagocytic phenotype, leading to BBB impairment [70]. This mechanism might also underlie the IBD-associated BBB disruption. Analyses of capillary-associated microglia (CAMs) unveiled that they are located around blood vessels, and their interactions primarily involve the microglial somata [71]. These interactions with capillaries are, in part, controlled by purinergic P2RY12 signaling. Microglia elimination by pharmacological treatment resulted in an increase in capillary diameter and cerebral blood flow and a decline in vasodilatative responses [71]. More investigations are needed to pinpoint how gut inflammation can affect the function of CAMs, which leads to vasculature dysfunction.

Microglia have prominent interactions with astrocytes, affecting their morphology and phenotype following different types of neuroinflammatory insult [72]. This interplay might have an important role in astrocyte abnormalities in depression studies. It has been previously shown that in the anterior cingulate, the white matter of depressed suicide astrocytes is hypertrophic [73]. Astrocytes play a vital role in maintaining BBB integrity. Under normal conditions, they release substances such as SHH; retinoic acid (RA); trophic factors, such as vascular endothelial growth factor (VEGF); and gliotransmitters like glutamate (Glu) that support new blood vessel formation and enhance endothelial cell junction tightness [74]. In response to inflammation, astrocytes increase SHH and RA secretion to counteract inflammation-induced damage. However, substances like VEGF and Glu may contribute to junctional damage, increasing BBB permeability. Elevated cytokine and chemokine secretion further facilitates BBB leakage and leukocyte migration [74].

These findings imply that different mechanisms can impact BBB integrity following gut inflammation and that microglia activation following IBD can affect the integrity of BBB via its interaction with astrocytes (Figure 1). It is worth mentioning that our knowledge is still not extensive regarding the effects of different experimental models of IBD on BBB integrity, and more investigations should be granted to study the effect of gut inflammation on BBB structure in different brain regions.

## 4. IBD-Associated Neuroinflammation—The Role of the Brain’s Innate Immune Response

Ample evidence now suggests that the cytokines produced during peripheral inflammation trigger a secondary, mirror inflammatory cascade in the brain, typified by microglia activation and the generation of proinflammatory cytokines, including TNFα, IL-1β, and IL-6 [75,76,77]. Electrophysiological changes in the hippocampus, a limbic structure involved in emotion regulation and cognitive function, have been reported in experimental IBD [78]. The notable dysregulation of the glutamatergic system due to alterations in the properties of postsynaptic α-Amino-3-hydroxy-5-methyl-4-isoxazolepropionic acid (AMPA) and N-methyl-D-aspartate (NMDA) receptors in acute colitis has been observed [79]. Specifically, the GluR2 subunit of AMPA receptors and the NR2B subunit of NMDA receptors were both downregulated [79]. Interestingly, there is substantial evidence that alterations in excitatory synapses may underlie depression [80]. Microglia-mediated inflammatory responses within the hippocampus during intestinal inflammation resulted in the enhancement of glutamatergic synaptic transmission and decreased synaptic plasticity, which may contribute to the behavioral comorbidities observed in IBD patients [79]. Furthermore, treatment with the microglia activation inhibitor minocycline, at doses that do not affect colonic inflammation, effectively abolished both increased TNF-α levels and the altered field potential and synaptic plasticity [79].

Both clinical and experimental investigations indicate that cytokines have the potential to elevate CNS excitability, subsequently leading to increased susceptibility to seizures, which has also been confirmed in murine colitis [81]. Intestinal inflammation altered the susceptibility to pentylenetetrazole-induced seizure in rodents [82], while further exploration unveiled a notable inflammatory reaction within the hippocampus, characterized by the activation of microglia and elevated levels of TNF-α, which mediated an increase in CNS excitability [26].

Sickness behaviors, including fatigue, mood alterations, and cognitive impairments, are frequently present in systemic inflammatory disorders [83]. While microglia, as the resident brain macrophages, are primarily implicated in this phenomenon, the connection between inflammation in peripheral organs, the signaling of circulating cytokines, and microglial activation remains inadequately understood. D’Mello et al. discovered that peripheral inflammation leads to an augmentation in monocyte-specific rolling and adhesion along cerebral endothelial cells (CECs) [21]. Signaling involving peripheral TNF-α and its receptor TNFR1, along with the adhesion molecule P-selectin, plays pivotal roles in facilitating these adhesive interactions between monocytes and CECs. These interactions were closely linked to the activation of microglia, decreased excitability in the CNS, and the development of sickness behaviors [21]. Investigating immune cell trafficking in the DSS-colitis model, Cluny et al. revealed that circulating monocytes, expressing α4β7 integrin, play a role in recruiting neutrophils to the cerebral blood vessels, resulting in higher levels of IL-1β and C–C motif chemokine ligand 2 in the brain of colitic mice, while the treatment with anti-α4β7 significantly reduced them. Elevated IL-1β, in particular, was identified as a mediator of anxiety-like behavior in this study [84].

The nitric oxide (NO) pathway is believed to play an important role in the neurobiology of depression [85] and also in the pathogenesis of depressive-like behavior associated with IBD [86]. Mice treated with TNBS displayed prolonged immobility duration in the forced swimming test, a significant increase in hippocampal TNF-α levels, the expression of iNOS, and nitrite content (Figure 1). The acute inhibition of NOS, with both specific and non-specific NOS inhibitors, resulted in reduced immobility time and decreased levels of TNF-α and nitrite content in hippocampal samples [86]. Dinitrobenzene sulfonic acid (DNBS)-induced colitis led to a decline in spatial recognition memory in the Y-maze task [87]. However, it did not influence the step-through latencies in the passive avoidance test. Memory impairment induced by colitis was counteracted with MK-801 or memantine, implying a role in disrupted NMDA receptor function as an underlying mechanism. Additionally, aminoguanidine inhibited colitis-induced memory deficits, suggesting the involvement of iNOS activation and oxidative stress in this phenomenon [87]. Although none of these investigations directly pinpointed the role of microglia in IBD-associated depressive-like behavior or memory deficit, it is well established that microglia are one of the major sources of TNF-α and NO following an inflammatory challenge [75,77]. More recently, a pioneer study demonstrated that DSS-induced colitis impairs spatial and recognition memory; activates microglia; increases A1-like astrocyte numbers; disrupts glymphatic clearance, worsening the accumulation of amyloid plaques; and causes neuronal loss in the cortex and hippocampus [88]. These neurodegenerative effects were linked to the increased expression of the NOD-, LRR-, and pyrin domain-containing protein 3 (NLRP3) inflammasome and the accumulation of gut-derived T lymphocytes along meningeal lymphatic vessels. Interestingly, the depletion of NLRP3 prevented cognitive impairment, neuroinflammation, and neurological harm triggered by colitis [88].

The involvement of the innate immune response in psychiatric disorders has been extensively explored through experimental and clinical studies [89]. In this context, microglia have emerged as a central focus of investigation [90]. Additionally, microglia are activated in the reaction relative to acute and chronic psychological stress [91,92]. This responsiveness of microglia to psychological stress could contribute to the underlying mechanisms of stress-related mental disorders, including major depression [93]. Importantly, stress has a significant impact on disease progression and exacerbations in individuals with IBD [22,94].

DSS-induced colitis led to a decrease in the immunoreactivity of Iba-1 and CD68 in certain regions of the limbic system, including the medial prefrontal cortex (mPFC) [95]. The most pronounced impact of colitis on microglia was observed in chitinase-like protein 3 expression, a microglial alternative activation marker. Conversely, the expression of another anti-inflammatory marker, CD206, decreased, while arginase 1 remained unchanged. As a result of colitis, elevated mRNA levels of CD86 and TNF-α were detected [95]. Peripheral inflammation can lead to long-term changes in the anterior cingulate cortex (ACC), a region of the brain known for its role in emotional regulation and cognitive processing, thereby mediating the development of mood disorders in IBD patients [96]. Within this brain region, the triggering receptors expressed on myeloid cells-1/2 (TREM-1/2), a family of cell-surface receptors highly expressed on the microglia, have emerged as potential modulators of the inflammatory and immune response. The overexpression of TREM-1 was observed in the ACC during the colonic inflammation phase, along with microglial and glutamatergic neuronal activation, while TREM-2 overexpression was noted in the remission phase and correlated with depressive symptomatology. Genetic and pharmacological manipulation downregulated TREM-2 expression and improved depression-like behavior [97].

Tally and colleagues demonstrated that mice treated with DSS exhibited weight loss, colon inflammation, and a notable increase in inflammatory cytokines in serum and brain, along with microglial activation [98]. The RNA sequencing of brains extracted from DSS-treated mice unveiled differential gene expression linked to the regulation of inflammatory responses, which returned to baseline levels after the discontinuation of DSS treatment [98]. It has also been demonstrated that colitis does not alter the number of microglial cells, but it does result in a higher count of monocyte-derived macrophages in the brain samples of animals with colitis [95]. These macrophages are either perivascular or enter the brain through the bloodstream, indicating an augmented infiltration of immune cells into the brain during colitis [95]. This suggests a potential link between increased immune cell infiltration due to inflammation and subsequent behavioral effects.

The depletion of microglia is an effective model for studying the regulatory function and activity of microglia. It is important to note that microglia depletion is not typically studied in the context of IBD. Methods for inducing microglia depletion include genetic inhibition [99] and also pharmacological inhibition using clodronate, a drug that mediates apoptosis, and it is used in an encapsulated manner in liposomes [100]. Highly specific inhibitors of colony-stimulating factor 1 receptor (CSF1R) are used to eliminate microglia, such as Ki20227, which demonstrated that the microglia-mediated increase in neuronal excitability was responsible for the psychomotor behavioral effects after local inflammation [101]. Furthermore, PLX3397 [102] and PLX5622 [103], also specific inhibitors of CSF1R, showed promising results for microglia depletion, affecting behavioral responses and gut–microbiota composition.

At the cellular level, the activation of microglia, along with astrocytes, and infiltrating blood-derived immune cells have been suggested as the potential initiators of neuroinflammation associated with IBD (Figure 1). Besides the temporal aspects of neuroinflammation in experimental colitis, it is also essential to consider a region-specific response within the CNS. Neuroinflammation induces neuronal dysfunction through distinct mechanisms, consequently contributing to behavioral abnormalities in IBD. While many of these potential mechanisms have been elucidated in the animal models of IBD, a significant limitation in most of these studies is the absence of a definitive causal link between concurrent neuroinflammation and the manifestation of depressive and anxiety-like behavior.

## 5. Synaptic Pruning Deregulation in Behavioral Comorbidities in IBD

Microglia, once considered solely as immune cells activated during inflammation or injury, are now recognized as key players in various aspects of normal brain functioning throughout developmental and adult stages. Multiple lines of evidence now substantiate the concept that microglia have a significant role in processes like neurogenesis and synaptic pruning, even in the absence of any inflammatory or immune-related stimuli [104].

One of the most investigated topics in microglia biology is synaptic pruning, the strategic elimination of synapses with reduced activity during the precise and critical stages of neuronal development. Microglia’s physiological roles are indispensable for the establishment and preservation of healthy neuronal circuits [105,106]. Different mechanisms involved in this process have been suggested in recent years. The complement protein C1q acts as a marker for synapses that need to be removed. Microglial cells engulf presynaptic inputs in early postnatal days when the phagocytic activity of microglia is high. The response is mediated by the complement receptor 3 (CR3) expressed by microglia [107,108]. Excessive microglial phagocytosis mediated by the complement system in adulthood resulted in the engulfment of excitatory synapses and reduced connectivity in the cortex, leading to behavioral abnormalities associated with stress [109]. Microglial synapse elimination also occurs through the fractalkine receptor (CX3CR1) [110]. TREM2 is essential for microglia to eliminate excessive synapses in the developing brain [111]. Adult mice lacking TREM2 exerted lower microglia activation during early brain development, sociability impairment, and altered brain connectivity [111]. Phosphatidylserine exposed to on neurons functions as a neuronal signal that prompts microglial-mediated pruning [112]. Yet, the specific molecular elements within neurons that determine which synapses should be removed remain unidentified.

Our knowledge about the possible effects of gut inflammation on microglia-mediated synaptic elimination in different brain regions and time periods is still scarce. Several studies have implicated that synaptic pruning might be impaired in different psychiatric paradigms [106,113]. Sellgren and colleagues demonstrated the enhanced removal of synapses in patient-derived neural cultures and isolated synaptosomes and observed irregularities in both microglia-like cells and synaptic structures [114]. Astrocyte-microglia IL-1R/C3/C3aR activation causes abnormal synaptic pruning and has an important role in mediating depressive-like symptoms in mice exposed to unpredictable chronic mild stress [115].

It is intriguing to note that the disruption of synaptic pruning could potentially serve as a connection between imbalances in the gut microbiota observed in IBD and certain neuropsychiatric conditions [44]. The enhancement of intestinal microbiota composition via dietary changes, probiotics, or fecal microbiota transplantation might contribute to better outcomes in neuropsychiatric disorders. This improvement could partially be attributed to the regulation of synaptic pruning and neuronal connections [44].

Autophagy, a process of cytosolic component and organelle degradation, has recently been discovered to regulate spine pruning in the mouse cortex, pointing out that autophagy might underlie the regulation of microglia-mediated synaptic pruning in IBD [116]. The selective deletion of the atg7 gene, which is essential for autophagy, in microglia leads to noticeable social behavioral impairments and repetitive behaviors—distinctive traits of autism spectrum disorders. This genetic intervention has also resulted in an increase in dendritic spines and synaptic markers, as well as changes in the connections between different brain regions, indicating alterations in the synaptic refinement process [117].

Gender differences are well established in the prevalence and clinical manifestations of IBD, along with depressive and anxiety disorders [118]. Sex hormones, such as estrogen, have known immunomodulatory effects, and fluctuations in these hormones may affect the immune response in both the gut and the CNS [119,120]. Considering the significant role of estrogens in shaping neuronal circuitry during the sexual differentiation of the CNS [121], it is important that future studies not only investigate the influence of estrogens on microglia-mediated synaptic pruning but also explore how these hormones affect the trophic and repair capabilities of microglia despite the well-documented neuroprotective effects of estrogen [122]. To investigate sexual dimorphism in microglial responses under intestinal inflammation, advanced techniques, such as single-cell RNA sequencing, proteomics, and translating ribosome purification analyses, should be used for a more comprehensive understanding of molecular mechanisms.

The complex and context-dependent roles of microglia in synaptic pruning involve various signaling pathways that can be of significant importance in the context of behavioral abnormalities observed in IBD. The role of autophagy in regulating synaptic pruning adds another layer of complexity to the understanding of these processes. Further research is needed to elucidate the connections between synaptic pruning abnormalities, microglial dysfunction, and the development of psychiatric conditions in the IBD context.

## 6. Neurogenesis Deregulation in Behavioral Comorbidities in IBD

**Figure 2:**
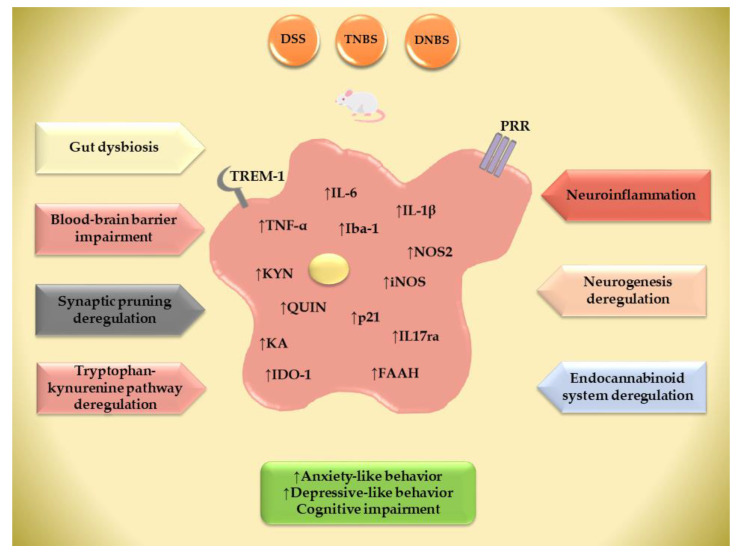
Summary of the main pathogenic mechanisms and their impact on microglia with consequential behavioral abnormalities from animal models of colitis. DSS—dextran sulfate sodium; DNBS—dinitrobenzene sulfonic acid; TNBS—2,4,6-trinitrobenzene sulfonic acid; Iba-1—ionized calcium binding adaptor molecule 1; IL-1β -interleukin-1 beta; IL-6 -interleukin-6; TNF-α—tumor necrosis factor alpha; iNOS—inducible nitric oxide synthase; NOS2—nitric oxide synthase 2; p21—cyclin-dependent kinase inhibitor protein; KYN—kynurenine; KA—kynurenic acid; QUIN—quinolinic acid; IDO1—indoleamine 2,3-dioxygenase 1; FAAH—fatty acid amide hydrolase; TREM-1-triggering receptors expressed on myeloid cells-1; PRR—pattern recognition receptors. ↑—increased.

Zonis et al. showed that chronic intestinal inflammation suppresses hippocampal neurogenesis [123]. Increased expressions of Iba1, a marker of activated microglia, IL-6, TNF-α, and the cyclin-dependent kinase inhibitor p21 (p21) in the hippocampus were detected in the acute phase of DSS-induced colitis, while the overexpression of p21 persisted in the chronic phase. Additionally, indicators of stem cells and early progenitor cells, such as nestin, brain lipid binding protein, and the neuronal marker doublecortin, were decreased. Conversely, the expression of glial fibrillary acidic protein, a marker of astroglial cells, was upregulated [123]. These findings imply that persistent intestinal inflammation is detrimental to the growth and development of neuronal precursor cells and may alter the properties and functioning of hippocampal circuits. In the acute phase of DSS colitis, enhanced neurogenesis and disruptions in the cell cycle of hippocampal progenitor cells were observed [124]. Chronic DSS colitis showed normal neurogenesis but impaired the migration and integration of new neurons into the dentate gyrus circuitry. Acute colitis increases the infiltration of peripheral macrophages and inflammatory myeloid cells into the hippocampus, leading to the increased expression of pro-inflammatory microglia and pro-inflammatory cytokines. In chronic colitis, higher proportions of tissue-repairing anti-inflammatory microglia were observed, along with elevated levels of the anti-inflammatory cytokine IL-10 [124]. Adult mice subjected to DSS during weaning displayed activated microglia, decreased hippocampal neurogenesis, and behavioral deficits [55]. Additionally, changes in neurogenesis were associated with the increased expression of pattern recognition receptors and Th17-cytokine receptors, indicating an immune response. Activated hippocampal microglia displayed morphological changes and increased the expression of Iba-1, IL1β, and NOS2 (Figure 2). These findings shed light on the significant effects of acute and chronic experimental colitis on adult hippocampal neurogenesis and innate immune cell responses [55,124]. They highlight potential mechanisms that may underpin cognitive and mood-related dysfunctions in IBD patients.

More cell-specific studies are needed to demonstrate the involvement of microglia in IBD-associated neurogenesis dysregulation: for example, using double transgenic mice that express microglia in one fluorophore and infiltrating macrophages in another one. Microglia depletion could also be used to investigate the relationship between the brain’s innate immune response and neurogenesis in IBD. It is also interesting to investigate the possible effects of antidepressants on neurogenesis abnormalities in the animal models of IBD.

## 7. Tryptophan–Kynurenine Pathway in Microglia and Its Possible Association with Behavioral Phenotypes in IBD

Recent research has revealed that various microglial phenotypes are linked to specific metabolic pathways, highlighting the crucial role of energy metabolism in shaping microglial functions. The imbalances in the tryptophan–kynurenine pathway have a significant contribution to the etiopathogenesis of mood disorders, which highlighted this pathway as a promising druggable target in psychiatric disorders [125]. The kynurenine pathway (KP), the primary route for tryptophan (TRP) metabolism, produces neuroactive metabolites, such as kynurenine (KYN), kynurenic acid (KA), and quinolinic acid (QUIN). The accumulation of these metabolites in the CNS is linked to neuropsychiatric diseases with inflammatory components [126]. Astrocytes and microglia are the main cell types within CNS that have the enzymatic machinery to metabolize TRP. The conversion of TRP to KYN by indoleamine 2,3-dioxygenase 1 (IDO1) constitutes the initial step in the KP. Under homeostatic conditions, KYN is mainly metabolized to KA by astrocytes. Microglia, on the other hand, regulate the KP by preferentially producing QUIN [126].

Post mortem findings on the role of TRP-KP in the pathogenesis of psychiatric disorders have been highly controversial [127]. Both increased [128] and decreased expressions of microglial QUIN [127,129] and reduced KP metabolism and cytokine expression [130] have been reported in individuals with depression. Elevated levels of CSF QUIN [131,132], reduced levels of CSF KA [132], and a higher ratio of QUIN/KA [131] were observed in psychiatric patients with a history of suicide attempts.

The potential of KP as a biomarker of response to antidepressant treatment has also been a subject of investigation, demonstrating that inflammatory biomarkers are associated with lower responses to this treatment [133]. Targeting KP with a particular focus on the principal rate-limiting enzymes, namely, IDO1, IDO2, tryptophan-2,3-dioxygenase (TDO), and kynurenine 3-monooxygenase (KMO), represents potential therapeutic strategy [126,134], although there is still a long way to develop cell-specific modulators of this pathway.

Recently, TRP-KP in glial cells has been introduced as a link between inflammation and mood disorders. It has been shown that IDO-1 gene expression is upregulated in the mPFC of mice with colitis [95]. Peripheral gut inflammation causes intestinal cells to release inflammatory cytokines, such as IFN-γ, IL-6, and IL-1. The activation of IDO by these inflammatory cytokines leads to an increased breakdown of TRP into KYN, which can cross the BBB and further be metabolized into various compounds, including QUIN and KA (Figure 2). In an inflammatory milieu, this augmented production of neurotoxic molecules, such as QUIN, 3-hydroxykynurenine (3-HK), and 3-hydroxy anthranilic acid (3-HAA), may potentially contribute to depressive symptoms by causing damage to hippocampal neurons [135]. DSS-induced colitis caused elevated KYN levels in the cerebral cortex, which were primarily a result of local synthesis mediated by the IDO-1, rather than transport from the bloodstream [136]. While there was no significant change in the pro/anti-inflammatory phenotypes transition of microglia cells in their response to colitis-induced KYN elevation, a neurotoxic neurotoxin subtype of astrocytes was observed. In addition, the changes in the variety and composition of the gut microbiota resulted in increased TRP metabolism in serum and brain [136]. When subjected to various forms of stress, experimental animals exhibit elevated ratios of KYN/TRP in both the brain and the intestines, along with an upregulation of IDO expression [137]. These stress-induced alterations are associated with changes in the composition and activity of the gut microbiota [138,139].

In the context of IBD-related inflammation impacting the TRP-KP in the CNS, significant emphasis should include the activation and expression of crucial TRP-KP enzymes like IDO, KMO, and KAT. A more in-depth examination of how TRP-KP intermediates are involved in CNS oxidative and antioxidative stress is also needed. Understanding these factors better can lead to the more precise management of inflammation and psychological conditions in IBD patients.

## 8. The Impact of Gut Inflammation on Central Endocannabinoid Function and the Development of Behavioral Comorbidities

The endocannabinoid system (ECS) plays an important role in modulating various emotional behaviors, including anxiety, primarily within specific brain regions like the amygdala, mPFC, and hippocampus [140,141]. The ECS comprises naturally occurring molecules, N-arachidonoylethanolamine (anandamide, AEA) and 2-arachidonoylglycerol (2-AG), and the enzymes responsible for their production and breakdown. The effects of ECS are orchestrated by cannabinoid receptors CB1 and CB2 [142]. Various studies have explored the link between peripheral inflammation, central eCB function, and the development of behavioral comorbidities [142,143].

The ECS has a significant impact in regulating microglial activity since microglia contain all components needed for a fully functional ECS and also produce enzymes that hydrolyze and deactivate AEA and 2-AG. CB1 and CB2 are known to be expressed by rodent microglia [144], while the presence of CB1 in human microglia is debatable [145]. These findings suggest that cannabinoids offer a promising target for influencing microglia function in pathological conditions. The CB2 receptor is not only essential for microglia activation triggered by Toll-like receptor stimulation [146] but also for activating the anti-inflammatory phenotype in microglia [147,148].

TNBS-induced colitis triggers anxiety and elevates circulating corticosterone, which is followed by the increased hydrolytic activity of the enzyme fatty acid amide hydrolase (FAAH), which breaks down the AEA in various corticolimbic brain regions [143]. Anxiety induced by colitis was alleviated via the acute inhibition of FAAH in the CNS, suggesting that the decrease in AEA played a role in the development of anxiety [143]. In the same colitis model, the inhibition of FAAH with URB597 increased the concentration of AEA in the colon and reduced its damage, improved survival rates, and preserved BBB integrity [149]. While cognitive function remained unchanged, the study suggests that modulating the ECS could be a potential therapeutic approach for IBDs and associated brain damage [149].

Notably, the human ECS has been implicated in depression pathology [150,151]. A meta-analysis by Kong et al. unveiled a strong link between the CB2rs2501432 polymorphism and depressive disorders, whereas no such association was found for the CB1rs1049353 polymorphism [152]. Furthermore, women with depression displayed notably reduced levels of AEA and 2-AG in their peripheral serum [153], while the increased expression of CB1 receptors was observed in the dorsolateral PFC of depressed suicides [154].

The role of ECS has also been studied in different stress paradigms [155,156]. A non-selective agonist of CB1 and CB2 receptors, WIN55,212-2, attenuated inflammation, anxiety, and stress sensitization in a repeated social defeat model. Reduced IL-1β mRNA was observed in the brain, but specifically in CD68+-activated microglia [155]. CB1 knockout (Cnr1^-/-^) mice were considerably more vulnerable to chronic social defeat (CSD) stress and mild CSD stress, exhibiting marked stress-related behaviors and increased microglial activity [157].

In general, it appears that the ECS protects against neuroinflammation by inducing anti-inflammatory profiles in microglia. Furthermore, diminishing the availability of eCB in the limbic area of colitis mice might be responsible for the induction of pro-inflammatory phenotype in microglia. However, studies with experimental models of IBD regarding ECS in the brain are very sparse. A considerable number of experimental and clinical investigations are needed to provide a novel perspective for the pharmacological management of psychiatric comorbidities in IBD via the modulation of endocannabinoid signaling.

## 9. Conclusions

Different lines of experimental evidence have indicated that chronic IBD can affect microglia function phenotypes. However, more cell-specific investigations are needed to address the mechanisms underlying microglia deregulation in IBD. This allows us to understand how microglia mediates IBD-associated behavioral changes. Furthermore, it is well established that spatial, temporal, and sexual heterogeneity exist in microglia function in health and disease conditions [32,35,37]. Hence, these heterogeneities should be considered when we study the effect of IBD-associated neuroinflammation and psychiatric comorbidities. It is highly possible that different brain regions, such as the limbic area, show different levels of microglia deregulation following gut inflammation.

The research area that focuses on targeting the activity of microglia in a time- and region-specific manner represents a great challenge. Nanotechnology holds the potential to address this challenge by facilitating the transport of large molecules through the BBB and facilitating the delivery of drugs [158]. Nasal applications have been successful in delivering pharmacotherapeutics and siRNA to target microglia in several animal studies [159]. Therefore, this approach might have an important translational value in clinical settings. Furthermore, microglia-specific genes can be targeted via the systemic administration of viruses that can pass BBB [160]. However, this delivery system has been tested only in animal studies. In addition, more mechanistic investigations should be carried out to address the effect of IBD on blood–brain barrier integrity and how microglia receive inflammatory signals from the periphery. Modulating microglia phenotypes by modifying inflammatory pathways may have important translational implications in alleviating IBD-associated neuroinflammation.
